# Advances in Sustainable Catalysis: A Computational Perspective

**DOI:** 10.3389/fchem.2019.00182

**Published:** 2019-04-12

**Authors:** Matthew G. Quesne, Fabrizio Silveri, Nora H. de Leeuw, C. Richard A. Catlow

**Affiliations:** School of Chemistry, Cardiff University, Cardiff, United Kingdom

**Keywords:** green chemistry, computational chemistry, density functional theory, QM/MM, homogeneous catalysis, heterogeneous catalysis

## Abstract

The enormous challenge of moving our societies to a more sustainable future offers several exciting opportunities for computational chemists. The first principles approach to “catalysis by design” will enable new and much greener chemical routes to produce vital fuels and fine chemicals. This prospective outlines a wide variety of case studies to underscore how the use of theoretical techniques, from QM/MM to unrestricted DFT and periodic boundary conditions, can be applied to biocatalysis and to both homogeneous and heterogenous catalysts of all sizes and morphologies to provide invaluable insights into the reaction mechanisms they catalyze.

## Introduction

The challenge of moving toward a greener and more sustainable society will inevitably require the drastic transformation of many aspects of modern culture and economy, with all areas of resource management and production needing radical overhaul (Liu et al., 2015; Little et al., 2016; Bakshi et al., 2018). From a green chemistry standpoint this means the reengineering of chemical pathways that: (i) make the most efficient use of natural resources (Hellweg and Canals, 2014; Bakshi et al., 2015, 2018; Jaramillo and Destouni, 2015), (ii) reduce the volume of hazardous/polluting reagents and solvents (Clark et al., 2015; Clarke et al., 2018), and (iii) promote the substitution of fossil fuel resources with renewable alternatives (Gallezot, 2012; Wettstein et al., 2012; Sheldon, 2014, 2016; Den et al., 2018; Talebian-Kiakalaieh et al., 2018). Achieving all these goals will require the design of novel and efficient catalysts that are active under mild conditions and can be produced sustainably without leading to unacceptably high levels of toxic pollutants (Beletskaya and Kustov, 2010; Polshettiwar and Varma, 2010; Chua and Pumera, 2015; Egorova and Ananikov, 2016). However, before any of these new catalysts can be developed a fundamental understanding of the properties of the currently most efficient and environmentally sustainable options has to be obtained, in order to enable the design of their replacement (Campbell et al., 2016; Hutchings et al., 2016; Pelletier and Basset, 2016; Friend and Xu, 2017; Chen et al., 2018; Kornienko et al., 2018; Caddell Haatveit et al., 2019). Computational models have proved to be one of the most efficient and least resource heavy ways of obtaining such information and have now become an invaluable component in the field as a whole (Nørskov et al., 2009; Hansgen et al., 2010; Medford et al., 2015; Sutton and Vlachos, 2015; Greeley, 2016; Grajciar et al., 2018). In recent years, joint experimental and theoretical catalytic studies have become routine and have proven crucial to any fundamental understanding of catalysis at the molecular level, which will be underscored in detail in the proceeding example sections of this perspective (Hirunsit et al., 2015; Van Speybroeck et al., 2015; Yu et al., 2017; Kulkarni et al., 2018; Zhu et al., 2018).

### Quantum Mechanical/Molecular Mechanics

**Figure 1:**
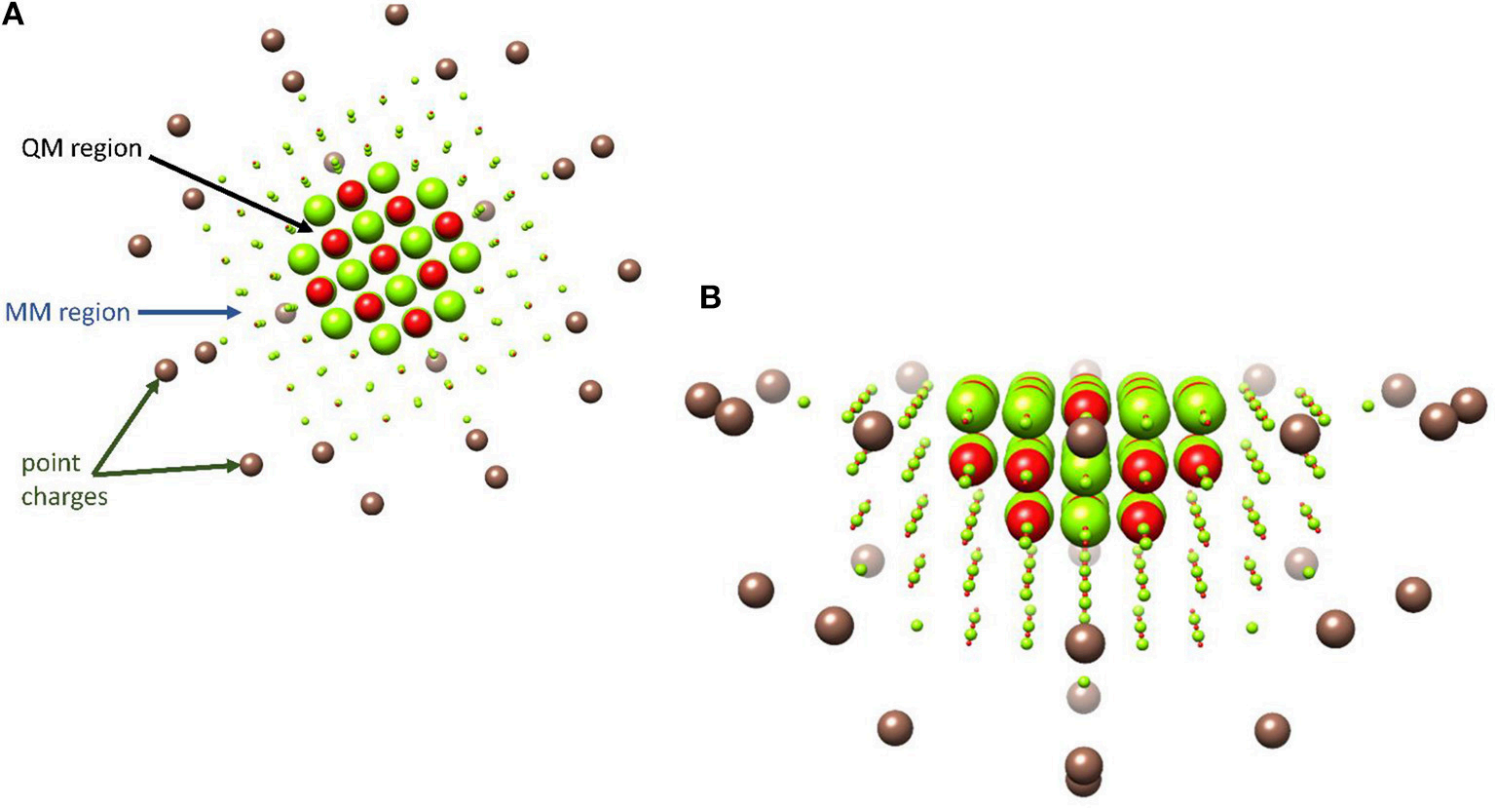
Illustrative representation of a typical QM/MM set-up of an example metal–oxide, MgO. A QM region with a circumference of 10 Å is shown with large red and green spheres, with point charges colored brown. **(A)** Top–view from above the slab, **(B)** side–on from the slab edge.

Computational chemistry really came of age during the 1960s, with the advent of mainframe computers; however, early breakthroughs in approximating the wavefunction of many electron systems date back almost 30 years earlier with the development of the Møller-Plesset second-order perturbation wavefunction theory (MP2) (Møller and Plesset, 1934). One major and much more recent development was the applicability of density functional theory (DFT), especially after the incorporation of the gradient approximations into the exchange correlation function (Becke, 1993). However, not all DFT functionals are created equally and at the turn of the millennium John Perdew proposed a climbing scale coined the “Jacob's ladder” with pure GGA functional near the bottom and hybrid functionals close to the top (Perdew and Schmidt, 2001; Sousa et al., 2007). In practice, this often means that *in silico* homogenous catalytic systems, which are often modeled with hybrid functionals (Green et al., 2014; Wójcik et al., 2016; Wojdyła and Borowski, 2016; Delarmelina et al., 2017; Dabral et al., 2018), produce results that are closer to experimental values then those obtained when modeling heterogenous catalysts, where pure GGA are frequently the only efficient functionals to be implemented periodically (Hammond et al., 2012; Zhao et al., 2015; Ishikawa et al., 2017; Kunkel et al., 2018; Morales-Gar et al., 2018; Fang et al., 2019; Wang et al., 2019). Unfortunately, there is no universal functional and the most appropriate exchange–correlation term must be assessed on a system specific basis by benchmarking theoretically obtained electronic or catalytic properties to those observed experimentally (Laurent and Jacquemin, 2013; de Visser et al., 2014; Hickey and Rowley, 2014; Cantú Reinhard et al., 2016); additionally, the amount of Hartree–Fock component included in the exchange component of the most commonly used hybrid functionals can be modified to produce a better match between experimental and *in silico* values (Reiher et al., 2001; Walker et al., 2013). Increasingly, the importance of systematically benchmarking the functional of choice to experimentally determined properties of heterogenous catalysts is also becoming widely understood (Janthon et al., 2013, 2014; Quesne et al., 2018; Zhang et al., 2018). Implementing such well-benchmarked quantum mechanical techniques, has led to an explosion in studies that highlight fundamental aspects of the reaction mechanisms catalyzed by (i) enzymatic biocatalysts (Meunier et al., 2004; Li et al., 2012; Quesne et al., 2013; Blomberg et al., 2014; de Visser et al., 2014), (ii) homogenous catalysts (Kumar et al., 2010; Prokop et al., 2011; Neu et al., 2014; Sahu et al., 2014; Yang et al., 2016), and (iii) heterogenous catalytic materials (Alfredsson and Catlow, 2002; Sun and Liu, 2011; Cadi-Essadek et al., 2015, 2016; Quesne et al., 2018; Schilling and Luber, 2018; Silveri et al., 2019). Moreover, when such techniques are combined with classical molecular mechanics or dynamics, hybrid QM/MM cluster models can be constructed that act as relatively computationally inexpensive methods for studying large catalysts from microporous and mesoporous materials (O'Malley et al., 2016; Catlow et al., 2017b; Nastase et al., 2019) to heterogenous nanocatalysts (Xie et al., 2017; Lu et al., 2019) and are often especially useful during the study of enzymatic reaction mechanisms (Gao and Truhlar, 2002; Senn and Thiel, 2007, 2009; van der Kamp and Mulholland, 2013). Indeed, one of the initial motivations for the development of the QM/MM method in the 1970s was to investigate such biocatalyzed reaction (Warshel and Levitt, 1976); although, despite the techniques early development, it was not until much later that the methodology really came into its own and the technique started to become widely applied (Field et al., 1990; Rothlisberger et al., 2000). As previously mentioned, QM/MM methods are now being increasingly used to model heterogenous catalysts, for example in modeling catalytic process in zeolites (Nastase et al., 2019) and supported nano-catalysts (Xie et al., 2017; Lu et al., 2019). An illustrative example of one such ionic catalyst is shown in Figure 1, where a QM region with a circumference of 10 Å has been applied to a slab of magnesium oxide.

Most QM/MM studies begin with a crystal structure that is often deposited and stored online, whereby database of everything from zeolites (Baerlocher and McCusker, 2010) to enzymes (Berman et al., 2000) as well as the primitive cells of ionic material (Hellenbrandt, 2014) are available to researchers. Heterogeneous catalysts tend to be very ordered with relatively small primitive cells that can be optimized using a 3D periodic scheme (Ghorbanpour et al., 2014); as incorporated in codes such as VASP (Kresse and Furthmüller, 1996a), CASTEP (Clark et al., 2005), and CRYSTAL (Dovesi et al., 2014), prior to the preparation of a larger supercell for QM/MM treatment. However, even with heterogenous catalysts, there may be a need for some post-optimization modification of the crystal structure to introduce defects and active sites; as is often the case with zeolites whereby Brønsted acid sites need to be included, which involves substitution of silicon atoms by aluminum and charge compensation by protons (Sastre et al., 2002; O'Malley et al., 2016; Nastase et al., 2019). Conversely, large catalysts with disordered tertiary structures often require a far more extensive preparation protocol, which is especially true for enzymatic catalysts, since it is often impossible to crystallize an active enzyme/substrate complex and heavy atom positions need to be modified to create reactant starting structures (Quesne et al., 2014). Additionally, all residues need to be protonated because hydrogen atoms do not have enough electron density to be resolved accurately; which is typically done to a specific pH value with a PROPKA server (Dolinsky et al., 2007). Finally, since most crystallization techniques reduce the water content inside the protein core, a detailed molecular dynamics protocol needs to be run to solvate the model using one of the biomolecular force fields designed specifically for proteins (Oostenbrink et al., 2004; Wang et al., 2004; Brooks et al., 2009).

Once a working model of the catalyst and substrate is obtained, the large system needs to be split into a minimum of two regions. Typically, the much smaller region contains all the atoms that need to be describe quantum mechanically (QM region) and the other much larger region contains all the remaining atoms (MM region); which are described at a much lower level of theory, often with classical molecular mechanics. Since creating these regions may necessitate the breaking of either covalent or ionic bonds, an accurate description of the interactions between different regions is critical for the correct electronic structure of the QM region. Currently the most common method for dealing with the valence issue in covalent systems, such as zeolites and biocatalysts, requires capping boundary atoms with hydrogen linkers (Senn and Thiel, 2009; Catlow et al., 2017a). When modeling ionic catalysts, interatomic potentials are often used to describe the MM region and an electrostatic embedding protocol is used to provide a countering polarizing environment to the ions at the border of the QM region (Bredow et al., 1996; Sokol et al., 2004).

Although, electrostatic embedding is also commonly applied to QM/MM models of covalent catalysts (Field et al., 1990) there is also the possibility of using a reduced mechanical embedding approach (Maseras and Morokuma, 1995). Both techniques model electrostatic interactions between atoms either side of the QM/MM boundary; however, MM charges are only included in the QM Hamiltonian with electrostatic embedding, which makes it the only appropriate methodology for ionic catalysts. In mechanical embedding protocols, electrostatic interaction between the two regions are assigned classically and so changes in the polarization of QM atoms due to electron transport (i.e., during a chemical reaction) is unaccounted for by changes in charge distribution (Chung et al., 2015). Importantly, the use of an electrostatic embedding protocol often produces results that are very sensitive to the choice of a given QM region, with convergence studies reporting that the absolute mean deviation between 40 different QM regions increased from 1.7 kcal mol^−1^ to ~5 kcal mol^−1^ when moving from a mechanical to an electrostatic embedding protocol (Hu et al., 2011). Therefore, the use of an electrostatic embedding protocol may lead to less accurate results for the study of covalent catalysts in cases where the boundaries of the QM/MM regions are chosen poorly. This problem is negated in for example QM/MM case study reported here, where ChemShell was used as a platform to create quickly create several different QM regions for benchmarking and the boundary regions were very carefully chosen to only cut through sp^3^ hybridized C–C bonds (Sherwood et al., 2003; Lu et al., 2019).

After a model of the catalyst is created, there are two major schemes for calculating the reaction landscape. Subtractive protocols are very commonly used in the study of reaction landscapes catalyzed by covalent catalysts, such as zeolites (Namuangruk et al., 2004; Vreven and Morokuma, 2006) and enzymes (Quesne et al., 2014; Wojdyla and Borowski, 2018). In two-layer subtractive protocols, only the QM region is capped with linker atoms because the whole system is also calculated at the MM level of theory, which would means that the MM energy of the QM region needs to be subtracted [*E*_*MM*_(QM_region_)] from the total energy to avoid double counting (see Equation 1). The QM/MM case study presented in this perspective utilizes the alternative additive approach, shown in Equation (2), whereby, only the MM region is calculated at the MM level of theory (*E*_*MM*_). This negates the need for a subtraction step but requires the addition of a specific coupling term to describe the QM/MM border region (*E*_border_), which includes bonding, electrostatic and Van der Waals interactions between the two regions (Sherwood et al., 2003). Importantly, when calculating using a mechanical embedding approach to calculate energy landscapes for covalent catalysts, it has been reported that both protocols should provide identical results (Cao and Ryde, 2018).

(1) $$\begin{array}{lll} E_{QM/MM}^{total} & = & E_{MM}^{whole}+E_{QM}\left(QM_{region}\right)-\;E_{MM}\left(QM_{region}\right) \end{array}$$

(2) $$\begin{array}{lll} E_{QM/MM}^{total} & = & E_{MM}+E_{QM}\left(QM_{region}\right)+\;E_{border} \end{array}$$

### Other Computational Techniques

One of the main alternatives to the QM/MM technique for the study of reaction mechanisms catalyzed by enzymes involves the use of QM cluster models that focus on the biocatalyst's active site region and immediate surroundings. The models can consist of dozens to hundreds of atoms, which are all treated with a highly accurate level of computational theory. Generally, the majority of the substrate binding pocket is included with priority given to charged hydrophylic residues that form strong hydrogen bonds or π-staking interactions with either the substrate or co-factors, which inevitably are also included (Siegbahn and Crabtree, 1997; Borowski et al., 2004; Hernández-Ortega et al., 2014, 2015; Miłaczewska et al., 2018). Thus, these models should faithfully mimic substrate position as well as the enzyme's catalytic activity; however, the need to add geometric constraints to these models can sometime restrict substrate mobility. Of course there are many advantages and disadvantages to both techniques, which have been well discussed elsewhere (Blomberg et al., 2014; de Visser et al., 2014; Borowski et al., 2015; Quesne et al., 2016a). Molecular cluster approaches have also been used successfully to calculate adsorbate energies and simulate frequencies for many heterogenous catalysts (Haase and Sauer, 1994; Pelmenschikov et al., 1995, 1998; Zygmunt et al., 1998; Dangi et al., 2010); however, the neglecting of long-range Coulomb interactions as well as the lack of realistic steric constraints can reduce the effectiveness of such techniques for calculating reaction pathways. Dynamical approaches such as metadynamics (Barducci et al., 2011; Qian, 2012), umbrella sampling (Kästner, 2011), transition path sampling (Bolhuis et al., 2002) as well as many others (Meliá et al., 2012; Roca et al., 2012), can also be applied to catalyst reactivity, where they can be very advantageous in the study of free energy landscapes and rare-events, which is especially true for large systems where there are many degrees of freedom to be considered along with many energetically close “representative” transition states (Tsai et al., 2002). Metadynamics aims to sample the three–dimensional free energy surface of a reaction landscape using one of several “collective” variable associated with the transfer atom(s) (Laio and Parrinello, 2002; Iannuzzi et al., 2003; Ensing et al., 2005; Laio et al., 2005) and has been extensively applied to zeolite (Moors et al., 2013; Van Der Mynsbrugge et al., 2014; Dewispelaere et al., 2015; Hajek et al., 2016; Cnudde et al., 2017) and enzyme (Petersen et al., 2009; McGeagh et al., 2011; Lira-Navarrete et al., 2014; Raich et al., 2016; O'Hagan et al., 2019) catalyzed reaction. Such techniques work best when a reaction coordinate can be assigned to a simple set of collective variables that apply to distinct groups inside the reactant(s); however, in cases where the reaction path is uncertain more degrees of freedom can be explored using a transition path sampling protocol. Such methods incorporate Monte Carlo techniques into a molecular dynamical algorithm to locate a number of potential transition states connecting different minima (Bolhuis et al., 2002; Petersen et al., 2009) and have also been extensively applied to both enzyme (Swiderek et al., 2014; Althorpe et al., 2016) and zeolite (Lo et al., 2005; Bucko et al., 2009) catalyzed reaction pathways. Of course, the holy-grail of modeling is to drive the first-principles design of these very large macro-catalysts from the ground up using knowledge about their functional building blocks and related existing catalysts to predict the three-dimensional structure of the whole *in silico*. Exciting developments in this field are being developed for both microporous (Wells and Sartbaeva, 2015; Nearchou et al., 2018) and biological catalysts (Zanghellini et al., 2006; Kiss et al., 2013) and aim to explore a much larger structural space than exists in the naturally occurring catalysts, opening up the potential for novel route toward sustainable chemical reactions (Muñoz Robles et al., 2015; Rodríguez-Guerra et al., 2018).

Neither QM/MM methods nor large restricted cluster model techniques are required for small homogenous catalysts, where a reasonable gas phase system can often be created using all of the catalyst and substrate atoms (Draksharapu et al., 2015; Sahoo et al., 2015; Greer et al., 2019). However, this is often not the case for the computational study of heterogenous catalysts, which are most commonly investigated using a periodic treatment to enable proper description of the band structure of a solid (Blöchl et al., 1994; Kresse and Furthmüller, 1996a,b). Notwithstanding the increased use of many of the advanced techniques as mentioned above, for these materials it is still extremely common to use periodic boundary conditions to simulate an infinite solid surface (Grau-Crespo et al., 2003, 2006; Janthon et al., 2013). It is also important to note that QM/MM approaches can be especially unsuitable for calculating metallic catalysts that have extended states that are not localized to and extend beyond the QM boundary. Examples of such systems are discussed in the final section of this perspective, whereby, the electronic properties and catalytic activity of various transition metal carbides are modeled in reciprocal rather the real space. The only cases where an unrestricted, molecular, DFT type protocol may be warranted is either when the number of atoms in the solid state catalyst are too few for banding to occur (Abuelela et al., 2012; Liu and Lee, 2012; Feng et al., 2018; Zheng et al., 2018), or when a specific geometric feature such as an edge site in a strongly ionic or covalent catalyst is under investigation (Pelmenschikov et al., 1996; Chieregato et al., 2014; Pasini et al., 2014; Geng et al., 2018). The example sections that follow, provide case studies where all these techniques have been applied to a wide range of different catalysts and work to highlight the potential for improving the sustainability of various chemical protocols by computationally led “catalysis by design.”

## Applications of Quantum Mechanics/Molecular Mechanics (QM/MM)

### Green Biocatalysis of Terminal Olefins From Fatty Acids

It is widely recognized that there is an urgent need for the development of sustainable replacements to crude oil (Kerr, 2007; Shafiee and Topal, 2009; Murray and King, 2012). Sustainable generation of bio-fuels utilizing biocatalytic pathways from fatty acid feedstocks has been identified as a promising area of research (Stephanopoulos, 2007; Kung et al., 2012; Peralta-Yahya et al., 2012; Straathof, 2014). However, much of these biosynthetic processes require whole cell techniques that reduce efficiency. Many of the alternative chemical synthesis protocols, used to transform fatty acids into terminal alkenes, are very far from green and require palladium catalysts and high temperatures (Gooßen and Rodríguez, 2004; Liu et al., 2014). In recent years, it has been reported that the bacterial P450 peroxygenases OleT_JE_ is able to catalyze the conversion of fatty acids to olefins without the need for additional cellular electron transfer machinery, since H_2_O_2_ and not O_2_ is used as the oxidant (Rude et al., 2011; Wang et al., 2014; Dennig et al., 2015; Grant et al., 2015). These medium-chain terminal olefins make excellent feedstocks for biofuels because they can be substituted for diesel without major engine modification and have improved temperature tolerance as well as a high energy content (Peralta-Yahya et al., 2012; Lennen and Pfleger, 2013). However, whilst such research does offer the possibility of an environmentally friendly route for the production of bio-fuels, at present, industrial application are limited by the abundance of side-products (alcohols). Therefore, before industrial applications can proceed there needs to be a more fundamental understanding into the origin of the bifurcation of the olefin and alcohol pathways. Two combined DFT and QM/MM studies have recently been published that investigate this bifurcation in depth, with the aim of steering bio-engineering of OLeT_JE_ to improve product selectivity (Ji et al., 2015; Faponle et al., 2016), and these studies will be discussed in our first example section.

**Figure 2:**
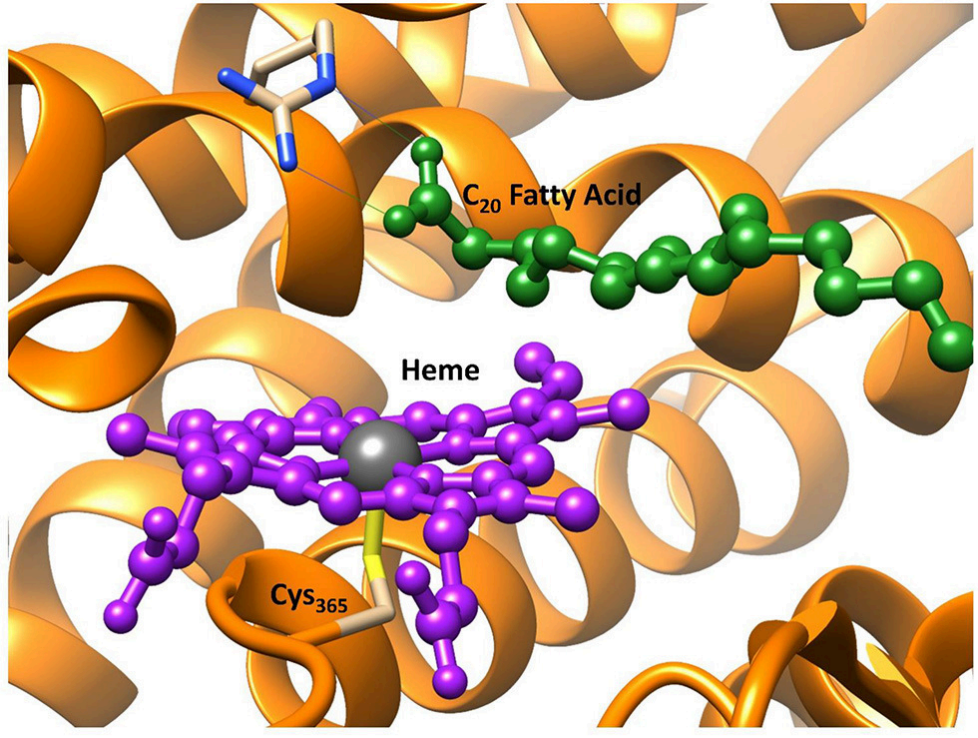
Active site region of P450 OleT, from PDB 4L40 (Belcher et al., 2014) with heme in purple and the fatty acid substrate in dark green. Figure modified using atomic coordinate reported previously (Ji et al., 2015; Faponle et al., 2016).

As mentioned above OleT_JE_ is a cytochrome P450, a family of enzymes that are ubiquitous and highly conserved throughout nature (Groves, 2003; Meunier et al., 2004; Ortiz de Montellano, 2004; Denisov et al., 2005; de Montellano, 2010; Kadish et al., 2010). Importantly, this enzyme family exhibits an extreme functional diversity in the reaction mechanisms they catalyze: from the metabolism of harmful drug molecules in the liver (Ji et al., 2015), to hormone biosynthesis (Guengerich, 2001; Posner and O'Neill, 2004; Munro et al., 2007) and they have also been commercially implemented in the cosmetics industry (Reinhard and de Visser, 2017). The active site region of OleT is depicted in Figure 2, and highlights the conserved thiolate linkage (Cys_365_) coordinated to an iron center of the heme co-factor, which are common features of all P450s (Poulos et al., 1985; Schlichting et al., 2000; Auclair et al., 2001). This resting state is primed to activate hydrogen peroxide via a hydrogen atom isomerization to form, the highly active iron(IV)-oxo heme cation radical species, Compound I (Cpd I) (de Visser et al., 2003; Shaik et al., 2005; Rittle and Green, 2010). Whilst there is significant structural homogeneity amongst the P450s, they often diverge in the residues close to their active sites; in general those enzyme that possess relatively tight binding pockets such as P450_cam_ oxidizing smaller substrate and those who incorporate more open active regions such as P450_BM3_ catalyzing larger substrate, like fatty acids (Gelb et al., 1982; Atkins and Sligar, 1987; Ruettinger et al., 1989; Davydov et al., 1999). In addition to the reduction in energy and toxic material consumption, many P450 isozymes demonstrate improved product regioselectivity over more conventional catalysts; therefore, their industrial application could lead to a reduced volume of wasteful side-products and therefore the biotechnological approach can be considered superior in terms of environmental sustainability (Grogan, 2011; O'Reilly et al., 2011). The question then concerns which aspects of OleT_JE_ causes its atypical product infidelity and can an in-depth computational investigation of its activity drive future bio-engineering of this enzyme toward selective bio-fuel production.

### Biofuel Production: What Drives Enzymatic Regioselective Toward the Olefin

**Scheme 1:**
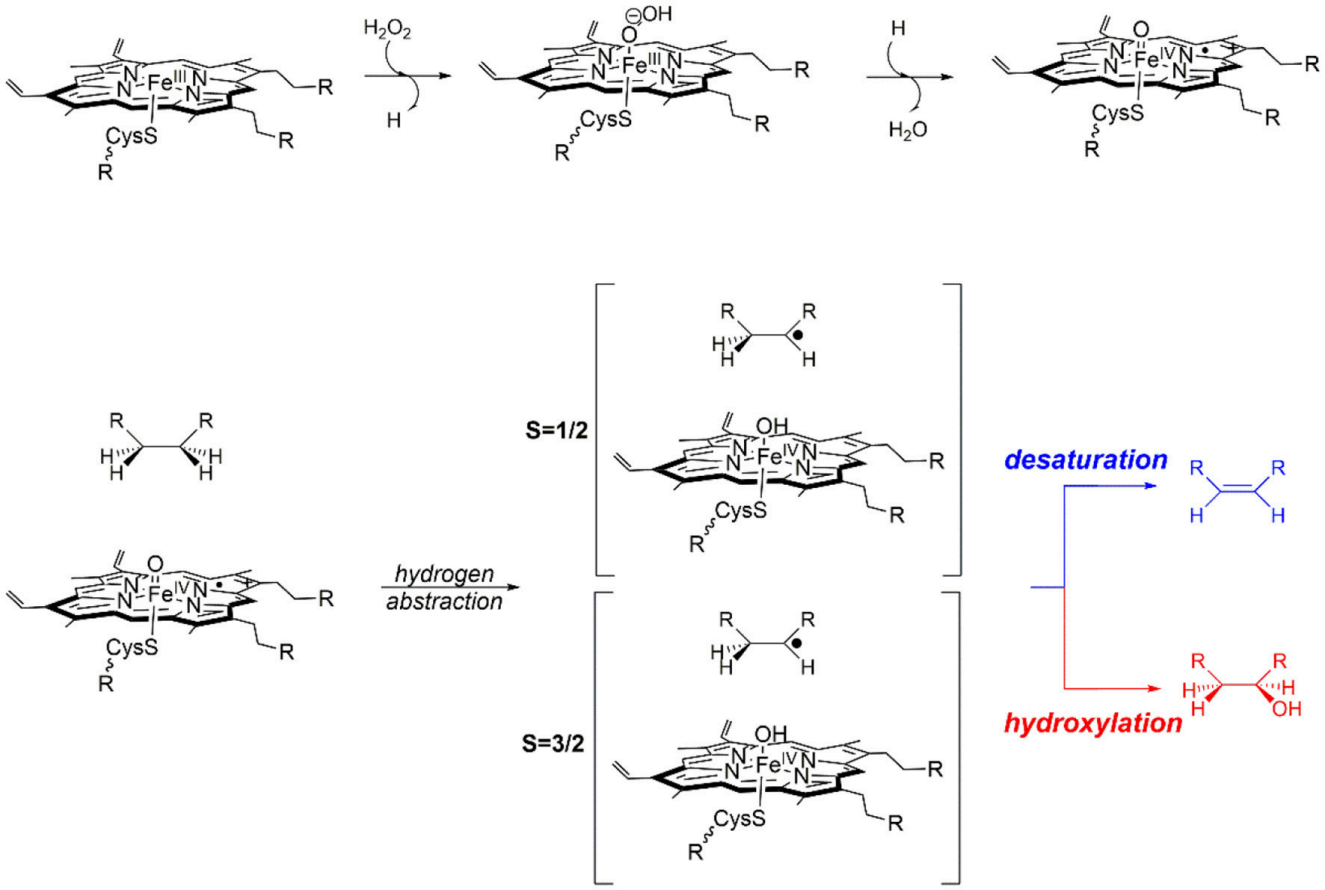
Proposed hydroxylation and desaturation pathways, as catalyzed by P450 OleT.

The bifurcated reaction mechanism proposed in Scheme 1, has been previously validated by computational models that predict the initial formation of a Cpd I species that exhibits spin-state selective product distribution (de Visser et al., 2001; Kamachi and Yoshizawa, 2003; Kumar et al., 2004b; Shaik et al., 2005; Quesne et al., 2016b). Computational studies that modeled only the first coordination sphere of Cpd I are in very good agreement with experimental observations (Rettie et al., 1987; Loch et al., 1995; Forkert and Lee, 1997; Sadeque et al., 1997; Lee et al., 1998; Wen et al., 2001; Gunes et al., 2007), whereby in general these models showed that Cpd I in the doublet spin state predominantly catalysis alcohol formation via small hydroxyl rebound barriers, whilst the quartet species can destabilize the radical intermediate and catalyze a broader range of products (de Visser et al., 2001, 2013). However, in order to confirm the veracity of this proposed mechanism for OleT_JE_ and to provide a deeper understanding of the effect of the protein environment on product specificity a combination of DFT and QM/MM techniques were required (Ji et al., 2015; Faponle et al., 2016).

These studies initially relied on small DFT models in order to evaluate the extent to which product specificity was driven by substrate properties, since *in vitro* experiments had shown that a member of this enzyme family catalyzes the exclusive hydroxylation of ethane (ET) to ethanol and the desaturation of dihydroanthracene (DHA), whilst valpronic acid (VA) can go through either pathway (Groves and McClusky, 1976). However, whilst the minimal DFT models did manage to predict the exclusive production of ethanol from ET, both DHA and VA showed similar reaction profiles, whereby, the doublet spin state catalyzed a combination of products via barrierless reaction mechanisms, whilst the quartet spin state catalyzed only the alcohol production via much lower hydroxyl rebound barriers. Therefore, the kinetic control exhibited by this active site model has proven insufficient for the understanding the different product selectivity reported for DHA and VA (Groves and McClusky, 1976). Importantly, this observation indicates that such a minimal model system may also be insufficient for the study of regioselectivity in product formation, as catalyzed by OleT_JE_. Excitingly, if product selectivity in these cases can be assigned to environmental factors remote from the first coordination sphere of the co-factor, then it may be possible to modify product selectivity through bio-engineering of OleT_JE_.

Therefore, to investigate the origin of the lack of fidelity in product regioselectivity vis-a-vis desaturation vs. α-hydroxylation of long chain fatty acids, as catalyzed by OleT_JE_, a detailed QM/MM protocol was initiated. The QM/MM model was designed starting from the crystal structure coordinates of the enzyme/substrate complex (see Figure 2) (Belcher et al., 2014). These crystal coordinates represent the enzymes resting state and therefore were modified to approximate the heavy atoms of the Cpd I active species, in a manner previously reported (Porro et al., 2009; Postils et al., 2018). Finally, the active enzyme/substrate reactant species was solvated, protonated, equilibrated and split into QM and MM regions before the reaction coordinates could be followed, using a well-established protocol (Kumar et al., 2011; Quesne et al., 2014). The QM/MM calculations employ a combination of the CHARMM27 force field (Brooks et al., 2009), as implemented in DL_POLY (Smith et al., 2002) and UB3LYP/SV(P) method as implemented in TURBOMOLE (Ahlrichs et al., 1989) with a solvent sphere of 35Å placed around the whole enzyme/substrate complex. All calculations were performed using the ChemShell code (Sherwood et al., 2003) as a platform to run an electrostatically embedded, additive QM/MM scheme. As hoped, this model did show that the presence of the protein environment had a major impact on product selectivity; whereby the ground state switched from the doublet, found in the small DFT model, to a quartet. More importantly, the product selectivity also flips with the decarboxylation barrier reduced from 17.8 to 5.1 kcal mol^−1^, which is below the 6.6 kcal mol^−1^ found for the hydroxyl rebound step. Thus, the ordering of the two barriers is reversed from that seen with the DFT model, where the alcohol production was favored by >10 kcal mol^−1^. A more detailed study found that this reversal in the barrier ordering was strongly dependent on the position of the hydrogen atom that was to be abstracted (Faponle et al., 2016). The energy barriers for hydrogen atom abstraction from the beta carbon of the fatty acid was very slightly lower than that seen with the alpha carbon. Importantly, hydrogen abstraction at the alpha position favored the alcohol production, whilst olefin production was dominant in the slightly more favorable beta radical intermediate. Intriguingly, while olefin production is favored using the QM/MM methodology, the two barriers are within the margin of error of the theory, which could help explain the mix product distribution seen experimentally (Rude et al., 2011; Wang et al., 2014,Dennig et al., 2015; Grant et al., 2015).

**Figure 3:**
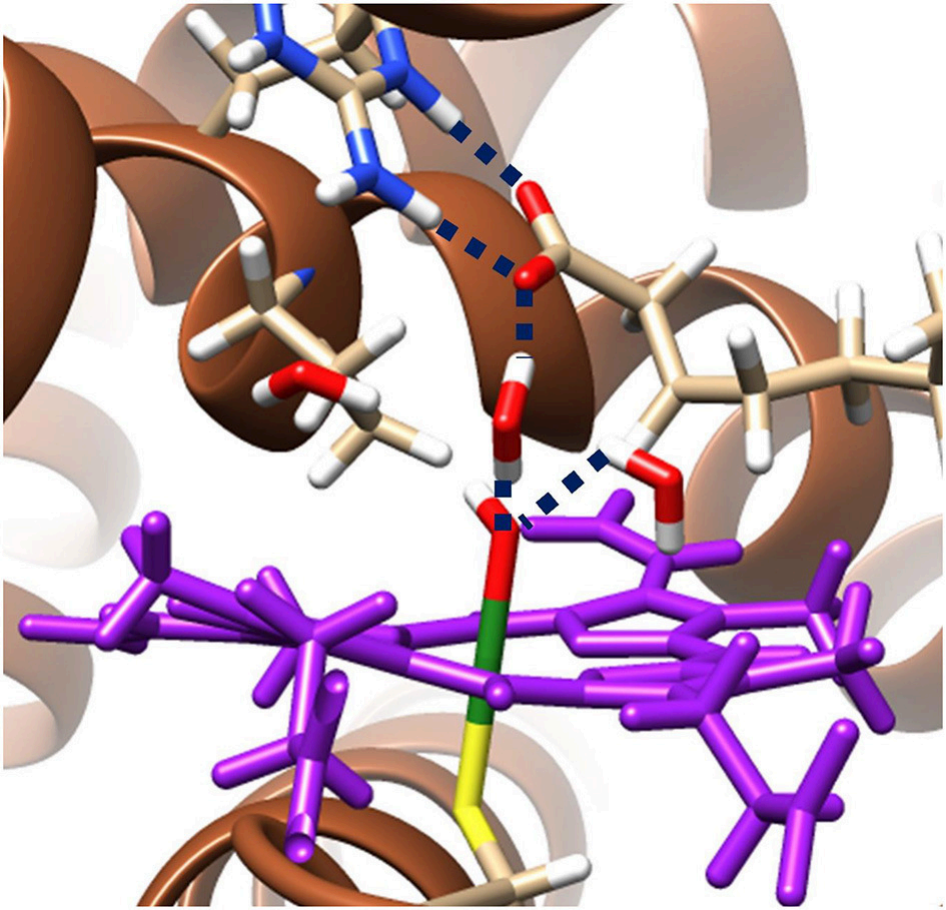
QM/MM model of the ground state radical intermediate of hydrogen atom abstraction catalyzed by OleT_JE_. Hydrogen bonding networks are linked with blue dashed lines and the heme co–enzyme is colored purple. The MM region is shown as brown secondary structure alpha–helices and beta–sheets; whilst, all atoms specifically highlighted are included in the QM region. Figure modified using atomic coordinate reported previously (Ji et al., 2015; Faponle et al., 2016).

The hydrogen bonding networks present in the ground state radical hydroxyl-intermediate are highlighted in Figure 3 and provide an insight into the origin of the reversal of product selectivity seen between the two methods. Often the use of a well-designed QM/MM protocol is the only way faithfully to replicate the local solvation environment surrounding an enzymes active region (Borowski et al., 2015; Quesne et al., 2016a). This phenomenon is evident here, with water networks surrounding the iron(IV)-hydroxo of the radical intermediate forming a bridge to a guanidine group of Arg_245_, which in turn increases the energy required for the rotation of the hydroxyl group, which is required to position the correct orbital overlap to initiate a rebound of the hydroxyl-group toward the substrate radical. Importantly, OleT_JE_ has an especially large binding pocket, allowing greater solvation of its active site. The effect of this is obvious when comparing the active site region of OleT_JE_ to other P450s such as P450_cam_, which tend to exclude much of the water from their active site (Poulos et al., 1987; Auclair et al., 2001). Thus, these results indicate that OleT_JE_ is able to effectively elevate the hydroxyl rebound barrier for radical recombination and therefore enable the pathway toward olefin production to become competitive. These results taken together are very encouraging with regard to the potential of directed bioengineering of a OleT based isoenzymes that is able to sustainably and selectively produce biofuels from terminal olefins, without the need for harsh reaction conditions or wasteful redox partners.

## Active Site Cluster Models

### The Promise of Sustainable Routes for the Catalysis of Spin Forbidden O_2_ Activation

Activating molecular oxygen in its triplet ground state is a very important step in many industrial processes (Wang et al., 2001; Liang et al., 2011; Suntivich et al., 2011). However, currently harsh conditions are generally required along with the use of a precious metal cofactor, which are in low-earth abundance and whose extraction has high environmental cost (Murthi et al., 2004; Zhang et al., 2007; Kotobuki et al., 2009; Widmann and Behm, 2014). This is due to the high stability and low reactivity of triplet O_2_, whose oxidation of substrates is often spin forbidden. Therefore, it is important to look at nature in order to develop more sustainable chemical pathway for oxygen reduction, which also drastically reduces the heavy metal component of the catalyst (Solomon and Stahl, 2018). However, most biocatalysts not only require metal cofactors but also organic co-enzymes, which in turn require the use of whole cell cultures to be regenerated, and these limitations reduce the utility of such enzymes in industrial processes (Solomon et al., 2000; Bugg and Ramaswamy, 2008; Quesne et al., 2015; de Visser, 2018). There is however, a small subgroup of these dioxygenases that are able to direct the spin-forbidden triplet to singlet conversion of molecular oxygen without the need of either a redox active metal co-factor or a sacrificial organic co-enzyme (Fetzner and Steiner, 2010). One of the few examples of this type of enzyme is the (1H)-3-hydroxy-4-oxoquinaldine 2,4-dioxygenase (HOD), which catalyzes dioxygenation of (1*H*)-3 hydroxy-4-oxoquinaldine (QND), leading to cleavage of the *N*-heteroaromatic ring (Bauer et al., 1996). Therefore, in our second example section, HOD was chosen as the subject of a couple of detailed studies (Hernández-Ortega et al., 2014, 2015), based on the DFT cluster model approach, into the basis of co-factor and flavin free activation of O_2_. It is anticipated that a detailed first-principles understanding of the origin of this activity could help direct the future design of industrial catalysts that can more environmentally perform spin-forbidden oxygenation reactions.

### Biocatalytic Activity of Metal-Independent Dioxygenases

**Figure 4:**
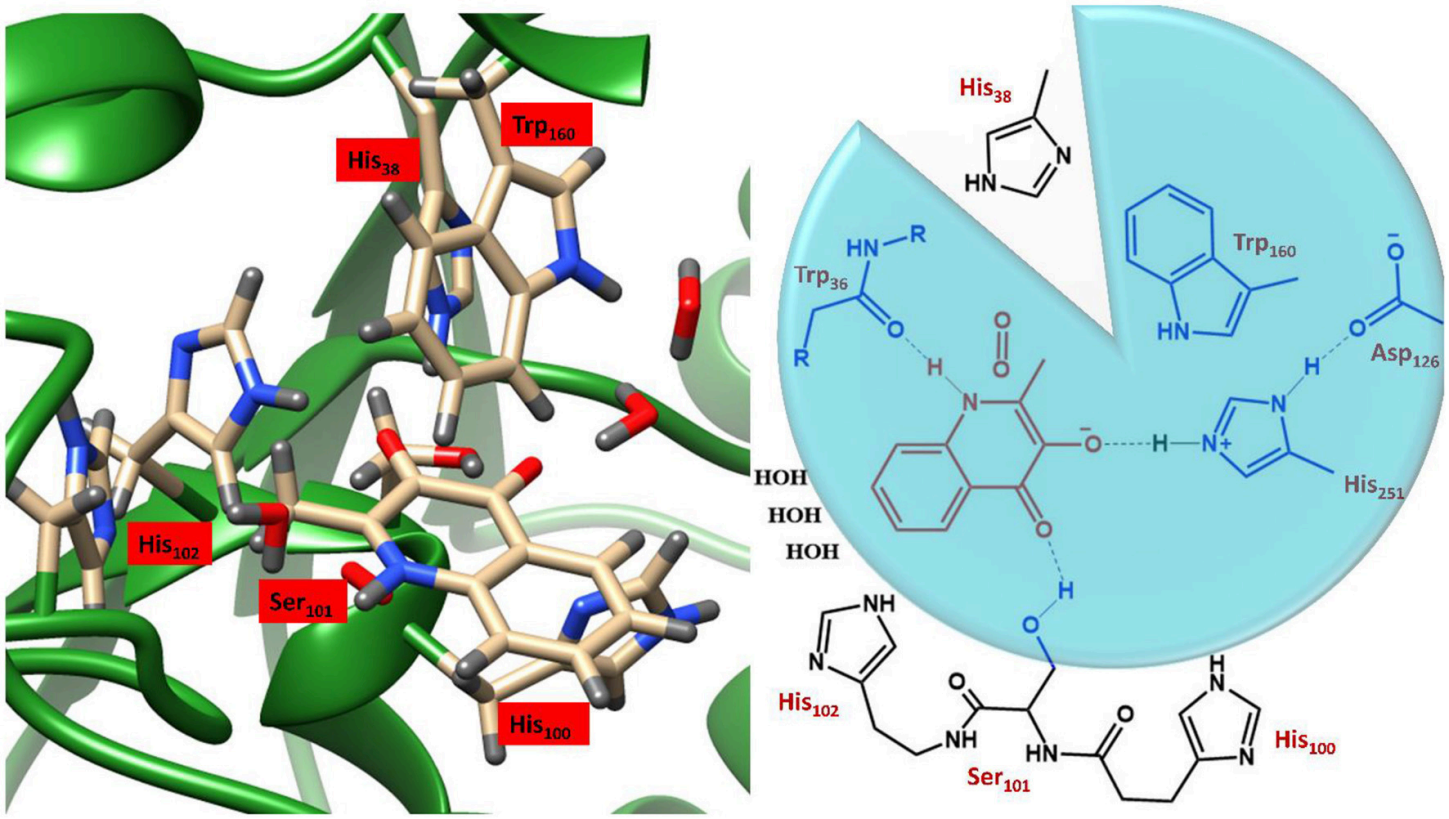
Illustration of the active site region of HOD and a schematic representation of the three DFT cluster models. Model A (red) simply contains the substrate and O_2_, Model B (blue circle) also the residues in blue, and Model B includes all residues depicted. Figure modified using atomic coordinate reported previously (Hernández-Ortega et al., 2014, 2015).

These studies employed variable sized DFT models, shown in Figure 4, where enzyme thermodynamics and kinetics were determined by models of only the substrate and molecular oxygen (highlighted in red). Of the two larger active site cluster models, the smaller one incorporated only the atoms in the blue circle, whereby, truncated versions of His_251_, Asp_126_, Ser_101_, and Trp_160_ and the backbone of Trp_36_ were added. The largest cluster model included all the atoms of the smaller ones as well as three water molecules and three additional imidazole groups, representing His_38_, His_100_, and His_102_ (black). Since the initial study focused purely on the formation of a substrate anion by a proton abstraction (Hernández-Ortega et al., 2014), oxygen was only present in the cluster models of the second study into the rate-limiting spin-forbidden activation of triplet oxygen, by the activated QND (Hernández-Ortega et al., 2015). The protocol for setting up the cluster models was very similar to that discussed in the previous section, whereby, the crystal structure was initially protonated, solvated, equilibrated and optimized. The crystal structure of the wild type enzyme was taken from the protein data bank (PDB) file 2WJ4 (Steiner et al., 2010), whilst the mutant variants were prepared *in silico* by modifying either a carboxylate or an imidazole group. As is shown in Figure 4, the QM regions of the two mutants lacked atoms for either a carboxylate or a carboxylate and imidazole group, for D126A and H251A, respectively. These structures were then equilibrated and optimized in the same manner described for OleT_JE_ (above), whereby a solvent sphere of 35 Å was placed around the whole system and the functional UB3LYP (Lee et al., 1988; Becke, 1993) in combination with the 6–31G(3d,p) basis set was used for the QM region. Therefore, whilst for this example QM/MM was not employed to investigate the kinetics of QND oxidation by HOD, it was used to obtain cluster model starting structures that represented protonated, solvated and optimized coordinates.

**Figure 5:**
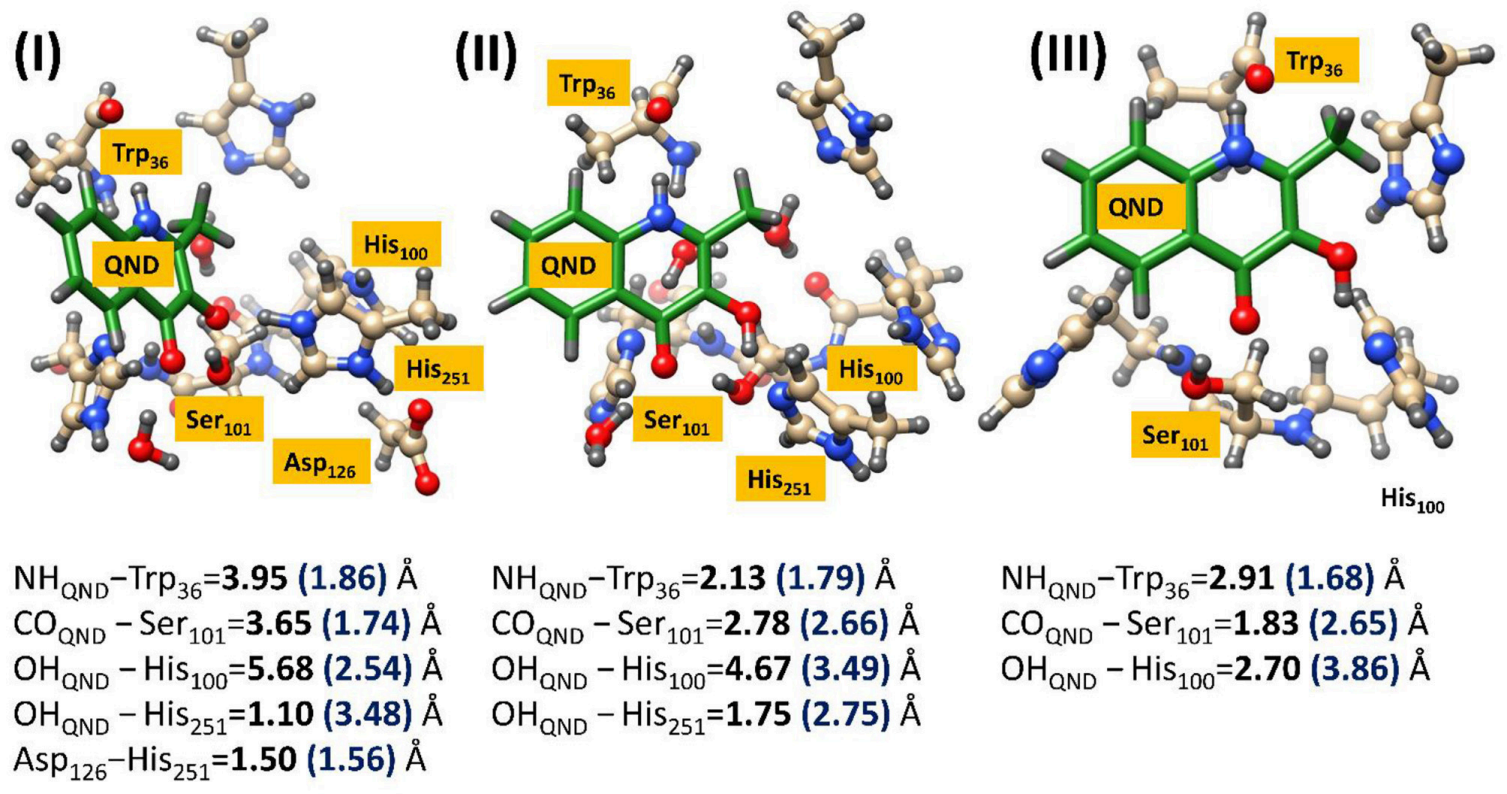
Comparison of the geometric changes between the initial QM/MM optimized structures and those obtained using a DFT cluster model, for three HOD variants. **(I)** Wild type HOD, **(II)** D126A, and **(III)** H251A. All systems were optimized at B3LYP/6-31+G^*^/Cluster//QMMM level of theory with important atomic distances from DFT (left) and QMMM (right) optimization given in Å. Figure modified using atomic coordinate reported previously (Hernández-Ortega et al., 2014, 2015).

This approach was required to obtain reasonable starting structures for the cluster models, which were also calculated using the same UB3LYP/6–31G(3d,p) methodology, only this time implemented in the Gaussian software package (Frisch et al., 2009). However, after the large cluster models were excised and optimized it became evident that the two technique gave radically different geometries, as can be seen in Figure 5. Much of these effects can be attributed to changes in substrate orientation that might to some extent be constrained by residues remote from the active site region. The precise position of the substrate seems to be significantly model dependent, although, there does appear to be a general migration away from hydrogen bonding networks with Trp_36_ and Ser_101_, in all models. Even though it could be argued that either increasing the cluster size or putting more constraints on the substrate might increase the match between the reactant geometries obtained by the two techniques, it was decided that such techniques would be too costly and could lead to unphysically high barriers along the reaction path. It is also important to note that the initial QM/MM optimized starting structures show there to be barely any effect of residue modification on substrate positioning, which may indicate that the QM/MM models are too inflexible to accurately simulate point-mutations. Alternatively, it is very possible that these two point-mutations would not be expected to produce a large amount of tertiary structure changes, and even if they did would require much more intensive molecular dynamics simulations to replicate *in silico*. In either case, as demonstrated below, it was shown that the cluster models were sufficient to provide important electronic insights into the origin of the experimentally observed differences in catalytic activity between the different variants.

The first study used a combined experimental and theoretical (cluster model) approach in order to investigate the preliminary proton abstraction step, which forms the active substrate anion. This combination technique underscored the importance of the histidine/aspartate dyad, since on its own His_251_ is not basic enough to abstract a proton from QND. Therefore, a strong hydrogen bond with Asp_126_ is required to catalyze QND deprotonation, which is evident by the >13 kcal mol^−1^ endothermicity of the smallest model shown in Table 1. These findings were replicated experimentally with the production of two mutant variants D126A and H251A, which each targeted one of the dyad residues with a point-mutation to an alanine. Each mutation caused a substantial drop in enzyme activity. Stop-flow experiments assessed deprotonation rate constants (k_H_) that were 5- to 40-fold lower in D126A and too low to measure in the H251A variant. Importantly, initial optimizations with both cluster and QM/MM models of wild-type HOD showed spontaneous proton transfer from the substrate; whilst, a stable hydrogenated QND species was found in both mutant clusters. The driving force for these different initial states is validated by the theoretical models, whereby, the wild-type models were the only ones to show exothermic deprotonation steps, with the thermodynamics of the D126A and H251A models pointing the equilibrium toward (QND)–OH (see Table 1). These substrate deprotonation energies report the bond formation energy of the His_(x)_–H, where His_100_ substitutes His_251_ in the H251A variant, minus the bond dissociation energy of the (QND)-H bond that is broken. As shown in Table 1, when these energies are calculated the slightly exothermic nature of the wild-type cluster model is set against endothermic energies of >10 kcal mol ^−1^ for D126A and ~30 kcal mol ^−1^ for H251A. Finally, it was determined that the origin of the loss in proton abstraction ability seen in the D126A variant was an increase in the proton affinity of His_251_ by 12 kcal mol ^−1^ upon coordination with Asp_126_.

**Table 1:** QND deprotonation by His residues, using active site models of different HOD variants and minimal models of the His/Asp dyad.

	**Δ*E* + ZPE**	**Δ*G***
WT	−0.9	−0.8
D126A	13.1	14.0
H251A	28.0	27.8
His251-H	13.4	13.3
His251-Asp126	1.8	1.8

*All Zero point (ZPE) and Gibbs free energies for QND deprotonation were obtained at UB3LYP/6–31G(3d,p) level of theory. All energies are given in kcal mol^−1^ and relate the deprotonated QND to reactant species*.

**Figure 6:**
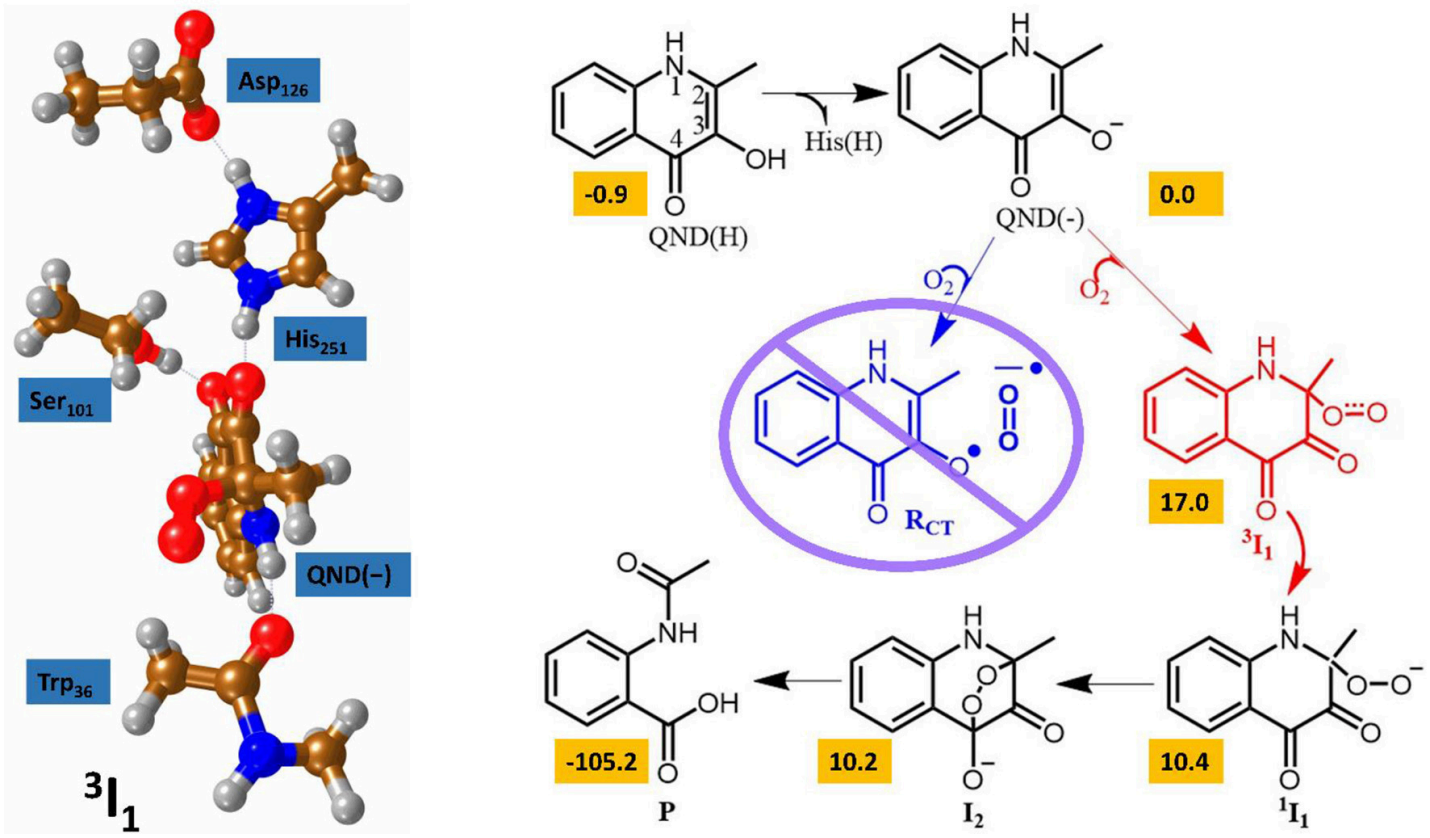
The two calculated reaction mechanisms for the oxidation of QND. The proposed pathway (blue) was assessed as less favorable than the alternative (red). A graphical representation of the alternative triplet intermediate is also shown (left). Figure modified using atomic coordinate reported previously (Hernández-Ortega et al., 2014, 2015).

At the time of publication, the computational results of the second study were somewhat controversial (Thierbach et al., 2014; Silva, 2016). This study focused on the spin-forbidden incorporation of triplet oxygen into the singlet product, the mechanism of which has wide implications for green chemistry. In this study the authors concluded that the rate-limiting oxygen activation step proceeded via an initial short lived oxygen bond triplet intermediate (Hernández-Ortega et al., 2015). Spin trapping experiments had been used to propose an alternative mechanism, whereby an initial long range electron transfer created a superoxo radical species that was then able to recombine with the substrate radical (see Figure 6) (Müller et al., 1987; Thierbach et al., 2014; Kralj et al., 2015). However, the authors of this theoretical study determined that their calculations indicated the experimentally observed radical could be more correctly assigned to the radical rearrangement that transformed the QND(–) substrate into the ^**3**^**I**_**1**_ intermediate with the aid of an elongation of the C2–C3 bond and the rehybridization of these two carbon centers from sp^2^ to sp^3^. The theoretical findings were additionally strengthened by transient state stop-flow experiments, which were completely unable to detect any signature that could have been assigned to the proposed **R**_**CT**_ intermediate. These studies provided a greater fundamental knowledge of this novel class of dioxygenases that could have important implications for future development of novel green catalytic routes to spin forbidden oxygen activation. These results indicate that the stabilization of a short-lived triplet intermediate, which last long enough for spin state crossing, could be key to future catalyst design.

## Homogenous Catalysts Modeled with Unrestricted DFT

### Oxidation of Methane to Methanol

Using density functional theory (DFT) methodology to characterize and rationally tune bioinorganic, earth-abundant and environmentally compatible homogenous catalysts, is a major field of combined computational and experimental research (Kumar et al., 2010; Prokop et al., 2011; Neu et al., 2014; Sahu et al., 2014; Yang et al., 2016). DFT techniques have been used to study the selective halogenation (Quesne and de Visser, 2012), nitrogenation (Timmins et al., 2018), and oxygenation (Jastrzebski et al., 2014) abilities of many such catalysts. Indeed, work on the selective dioxygenation of catechol by tris(2-pyridylmethyl)amine (TPA) has shown promise for the potential of a sustainable, green-catalytic route for nylon production via dimethyl adipate (Jastrzebski et al., 2013, 2014). Homogenous catalysts are often able to catalyze reaction mechanisms selectively at far lower temperatures and pressures then conventional routes. Indeed, selectivity can be one of the most important environmental benefits of choosing homogenous catalysts because of the increased yields and lower side-products. However, it is important to consider the potential of homogeneous catalysts increasing the volumes of contamination and waste, as well as the excess energy required for product separation and catalyst recycling, which is often greater than observed using heterogenous catalysts (Lam et al., 2010; Tan et al., 2013). Therefore, it is crucial to consider to what extent there could be an overall environmental benefit to using homogenous catalysis over the more conventional heterogenous routes (Corma and García, 2003; Astruc, 2007; Baroi and Dalai, 2015). The example catalyst presented here, is very novel for possessing the ability of converting methane to methanol at low temperatures and ambient pressures (Kudrik and Sorokin, 2008; Sorokin et al., 2008, 2010; Isci et al., 2009; Kudrik et al., 2012). Whilst the reactivity of this bio-mimetic catalyst has been well characterized, the origin of its efficiency was poorly understood. Therefore, a detailed an in-depth computational study was undertaken to understand the aspects of its catalysis that enabled such high activity toward methane hydroxylation, so that such a fundamental understanding of reactivity could be used to further improve the activity of this or related catalysts (Quesne et al., 2016b).

As discussed in the olefin production section, the P450 super family of enzymes are amongst the most efficient and powerful catalysts for oxidizing C-H bonds. However, despite the extreme amount of substrate diversity, there is no natural pathway that utilized a P450 isoenzyme and is also powerful enough to activate the 104.9 kcal mol ^−1^ strong C–H bond of methane. In fact, under guest/host activation conditions, methane was the only short chain alkane that P450_BM3_ was unable to oxidize (Kawakami et al., 2011, 2013; Zilly et al., 2011). Bioengineering does a little better than nature with CYP_153A6_ actually showing some oxidation activity toward methane, although, with an extremely slow turnover frequency of 0.02–0.05 (Chen et al., 2012). These observations are in stark contrast with the μ-nitrido-bridged diiron-oxo porphyrin catalyst discussed in this section, which has demonstrated high oxidative activity toward methane and is based on a dimer of two of the co-enzymes found in P450s (Kudrik and Sorokin, 2008; Sorokin et al., 2008, 2010; Isci et al., 2009; Kudrik et al., 2012). The active site of P450 enzymes consists of thiolate linked iron-oxo porphyrin (see Figure 1). Importantly, the observation that the use of perfluoro-carboxylic acid to enable alkane activation by P450_BM3_ proved insufficient for methane oxidation provides evidence against a mechanism whereby the lack of P450 activity is simply due to the absence of a isoenzyme that is able to accommodate methane in its active site (Kawakami et al., 2011, 2013). An alternative explanation for the inferior activity, toward the oxidation of methane, demonstrated by P450s over the diiron porphyrin catalysts is seen in the numerous studies into the effect of different axial ligands in the catalytic activity of iron-porphyrins (Gross and Nimri, 1994; Czarnecki et al., 1996; Song et al., 2005; Takahashi et al., 2012). Regardless of the relative importance of either of these affects, the origin of the massive improvement seen in the diiron porphyrin dimer above the mono-porphyrin bio-catalyst is of crucial important for the future design of the next generation of powerful oxidants for sustainable methanol production.

### Origin of the Catalytic Activity of Diion(IV)oxo Porphyrinod

**Figure 7:**
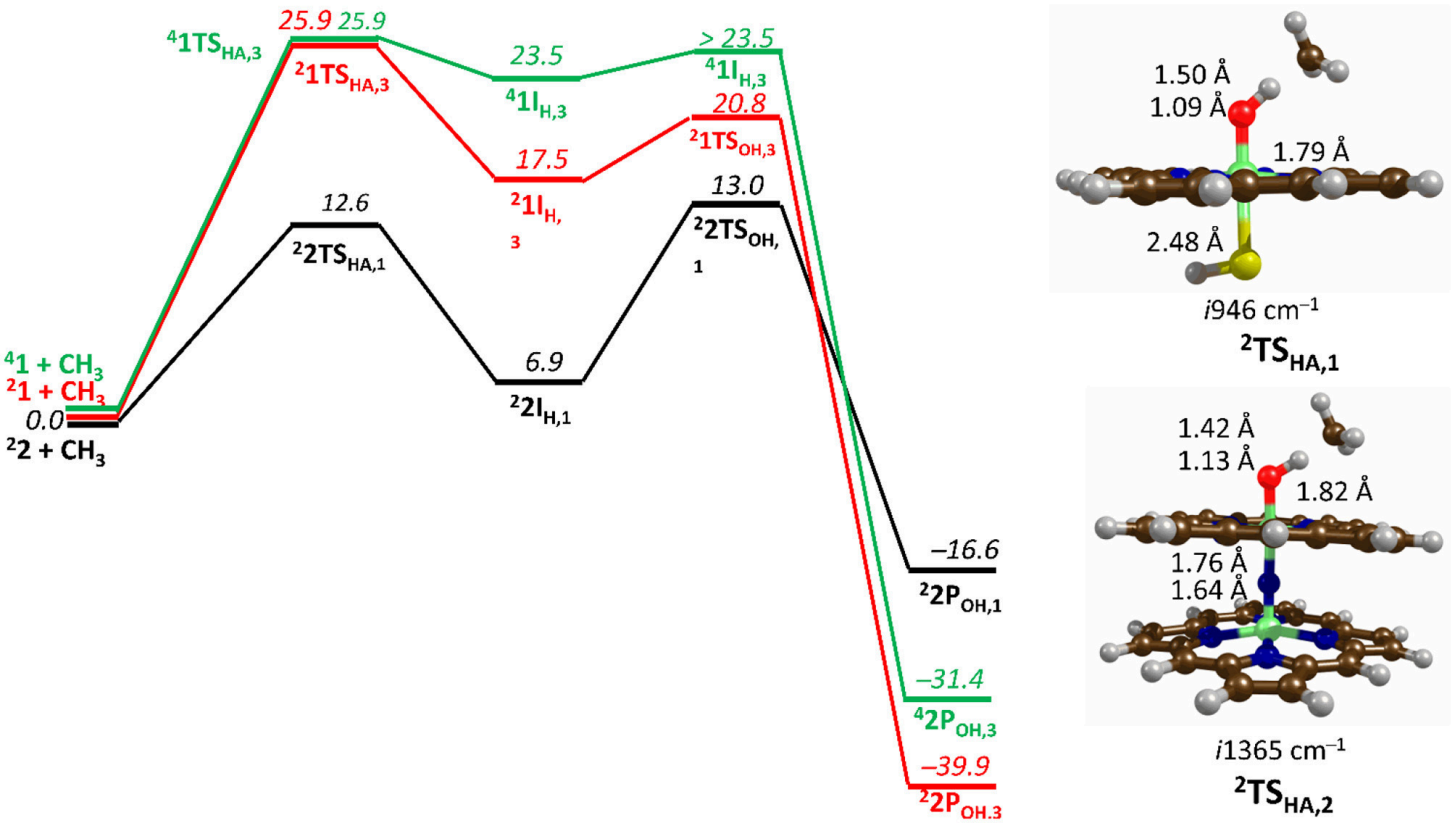
Free energy methane hydroxylation pathways as catalyzed by the μ-Nitrido-Bridged diiron porphyrin (**1**) and CpdI (**2**). Energies calculated at UB3LYP/BS2//UB3LYP/BS1 level of theory and given in kcal mol ^−1^ relative to the reactant species. Figure modified using atomic coordinate reported previously (Quesne et al., 2016b).

Over the years, a minimum model of Compound I (see **1**, Figure 7) has been extensively tested and proved to be sufficient for explaining the first coordination sphere P450s (Yoshizawa et al., 2001; de Visser et al., 2004; Shaik et al., 2005), which is the same model ([FeIV(O) (Por^+^) −SH]) that was mentioned in our first example section and consisted of an iron-porphyrin coordinated by an oxo group *trans* to an SH, representing the axial cysteine. The μ-nitrido-bridged diiron-oxo porphyrin model (see **2**, Figure 7) replaces the SH group for a nitrogen, which became a linker for a second iron-porphyrin. The DFT calculations were performed without any geometric restrictions on any of the atoms in the model systems and full optimizations were undertaken for each minima using UB3LYP (Lee et al., 1988; Becke, 1993), in combination with the initial double-ζ basis set 6–31G (Ditchfield et al., 1971) on all atoms except for Fe where LACVP with a Neon core potential was implemented (BS1). Subsequently, single point gas-phase and solvent corrected calculation were run using the same functional in combination with the polarizable and defuse triple-ζ basis sets 6–311+G(d,p) and LACV3P+ (BS2). Free energy values were calculated at 298.15K and 1 atmosphere of pressure, with such a protocol being well benchmarked previously (de Visser, 2010).

Figure 7 shows the free energy landscapes for the methane to methanol reaction as catalyzed by both model catalysts. Since the energy barriers associated with the hydrogen atom transfer (HAT) on both the doublet and quartet spin state surfaces are the same to the first decimal place for catalyst **1**, the energy landscapes of both are considered. This spin state derived bifurcation in the pathways catalyzed by CpdI is in excellent agreement with multiple different studies (de Visser et al., 2003, 2014; Kumar et al., 2004a,b) and stands in sharp contrast to the diiron porphyrin dimer catalyst (**2**), which exhibits a doublet ground state with a rate-limiting step that is well separated (by >40 kcal mol ^−1^) from the hydrogen atom abstraction barrier on the quartet energy landscape. Such a dominant doublet ground state is also in excellent agreement with previous work on **2** (Silaghi-Dumitrescu et al., 2011; Ansari et al., 2015; Işci et al., 2015). Therefore, only the low spin state surface of **2** is included in Figure 7. The reaction mechanism for both catalysts proceeds via a rate-limiting hydrogen atom abstraction transition state (**TS**_**HA**_) leading to a radical hydroxyl intermediate (**I**_**H**_), which is capable of forming methanol (**P**_**OH**_) through radical recombination with the methyl radical following a hydroxyl rebound barrier (**TS**_**OH**_). The decrease in the free energy barrier for **TS**_**HA**_ of 13.6 kcal mol ^−1^ for **2** over **1** corresponds to a rate enhancement of ~10^10^. Indeed, such a low barrier would imply that **2** was able to catalyze the oxidation of methane at room temperature, which has been observed experimentally (Sorokin et al., 2008). The bond length shown in Figure 7 show that the high energy **TS**_**HA**_ seen in **1** is considerably more product like with a shorter O-H and a longer C-H distance than is the case for **2TS**_**HA**_. Notwithstanding these differences, both transition states are generally product like, which was expected and is a consistent trend in methane hydroxylation barriers (Yoshizawa et al., 2001; de Visser et al., 2004; Shaik et al., 2008).

**Figure 8:**
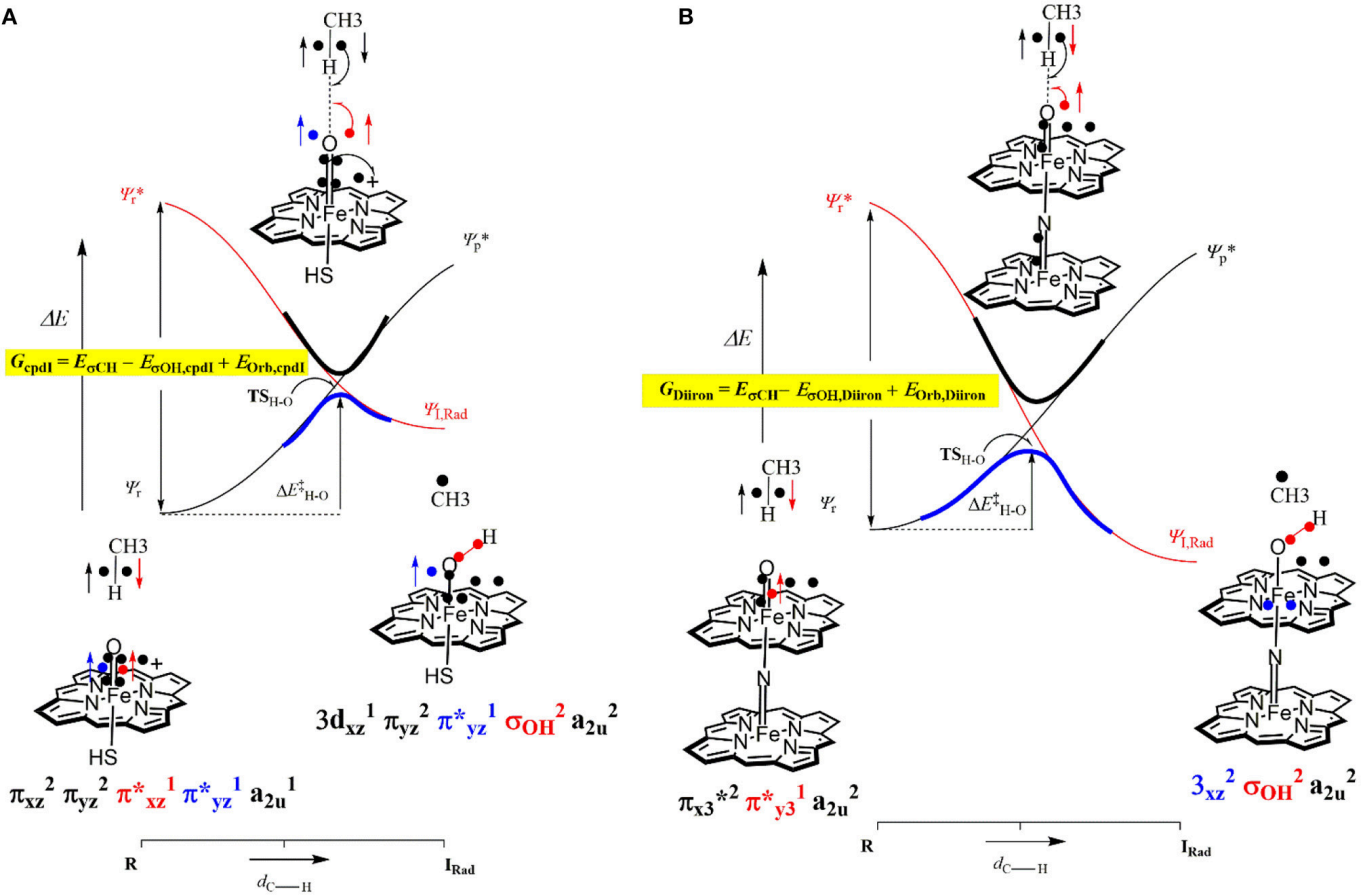
Valence bond diagram of methane hydroxylation by models of P450 CpdI **(A)** and the Diiron porphyrin catalyst **(B)**.

**Figure 9:**
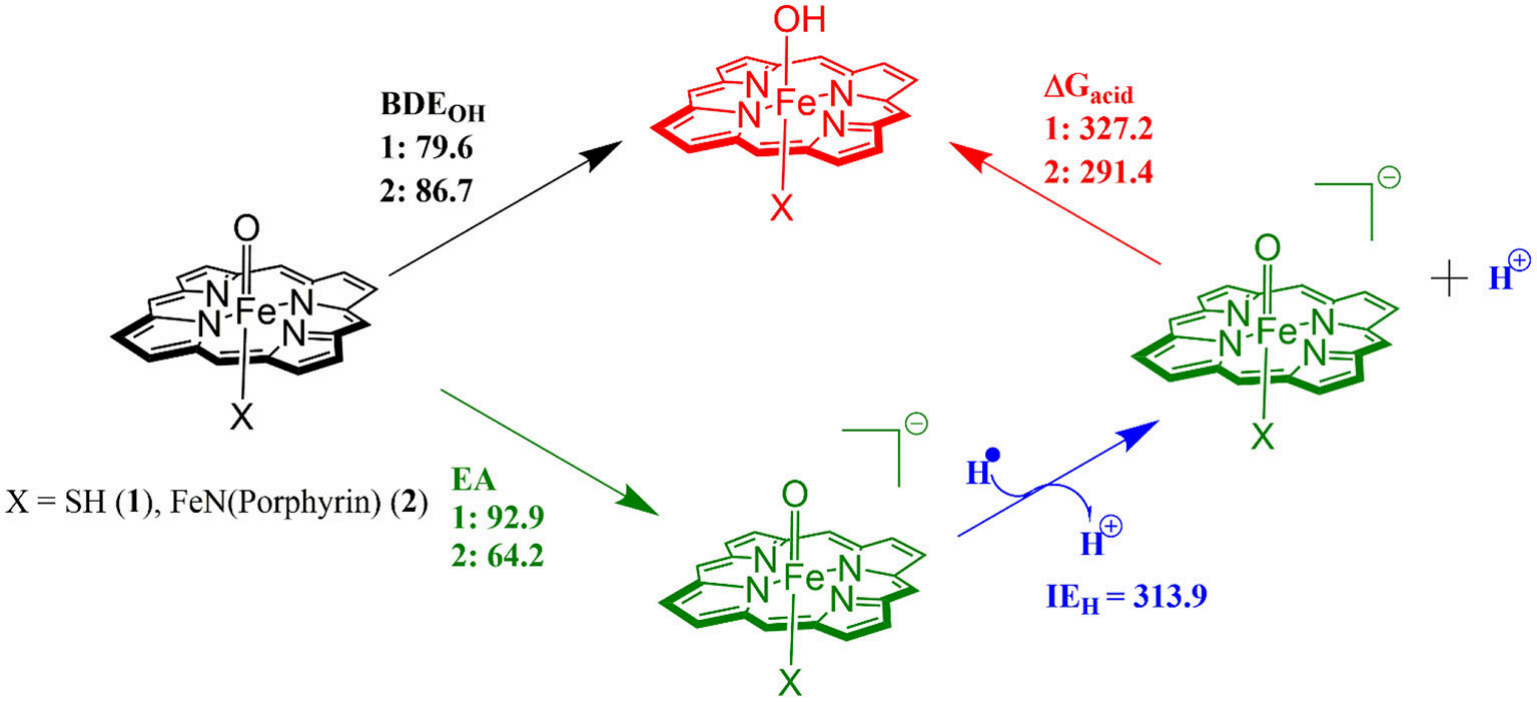
Theoretically determined free energy values for the hydrogenation of catalysts **1** and **2**. All solvent corrected free energies were obtained at UB3LYP/BS2//UB3LYP/BS1 level of theory and are given in kcal mol^−1^.

The divergence in the performance of these two catalysts can be explained by differences in the electronic structure with regard to the location of valence electrons, as shown in Figure 8. The valence electrons for CpdI (**1**) are shown on the left and have an occupancy of $\pi_{\text{xz}}^{2}$, $\pi_{\text{yz}}^{2}$, $\pi_{\text{xz}}^{*1}$, $\pi_{\text{yz}}^{*1}$ dominated by the Fe(IV)oxo combined with a singly occupied ${\text{a}}_{2\text{u}}^{1}$ on the heme cation radical. Therefore, CpdI has a ground state with a total of three unpaired electrons and the close lying doublet and quartet spin state only diverge electronically by either anti-ferromagnetically or ferromagnetically coupled heme and FeO orbitals (de Visser et al., 2003; Porro et al., 2009). In **2** the eight valence electrons of an axial Fe(IV)-nitrido mix with the seven of the Fe(IV)oxo and the energy of the a_2u_ orbitals of both porphyrins are lowered to give an occupancy of $\pi_{\text{x}1}^{2}$, $\pi_{\text{y}1}^{2}$, $\pi_{\text{x}2}^{2}$, $\pi_{\text{y}2}^{2}$, ${\text{a}}_{2\text{u},1}^{2}$, ${\text{a}}_{2\text{u},2}^{2}$, $\pi_{\text{x}3}^{*2}$, $\pi_{\text{y}3}^{*1}$. For simplicity only the two occupied anti-bonding π– orbitals as well as the highest lying a_2u_ orbital is included in Figure 8. In retrospect such a change is not unexpected since it has been determined that mixing between the 3P_Z_ orbital on the axial Sulfur atom, which is absent in **2**, and the a_2u_ porphyrin orbital raises the latter's energy above that of the π FeO orbitals and leads to the porphyrin radical in **1** (Ogliaro et al., 2001). However, the degree to which this change affects the electron and proton affinities of **2** is somewhat more surprising, since research has indicated that the kinetics of HAT reactions is usually correlated to the thermodynamics of hydrogen atom binding (**BDE**_**OH**_) (Friedrich, 1983; Bordwell and Cheng, 1991; Mayer, 1998). Figure 9 breaks down the **BDE**_**OH**_ of both catalysts into electron (**EA**) and proton (**Δ**_**acid**_) affinity components. Therefore, for each catalyst **BDE**_**OH**_ = **Δ**_**acid**_ – **EA** – **IE**_**H**_, whereby (**IE**_**H**_) describes the ionization energy of a hydrogen atom. It is clear from Figure 9 that whilst the electron affinity of the FeO is greater by ~ 29 kcal mol^−1^ in the CpdI mimic this is more than compensated by the >35 kcal mol^−1^ increase basicity of the anionic species of **2**, which is consistent with other studies that show the dominance of **Δ**_**acid**_ in **BDE**_**OH**_ (Green et al., 2004; Parsell et al., 2009). Therefore, it is the increase basicity of the μ-nitrido-bridged diiron-oxo porphyrin that is the origin of its increased activity and any attempt to further design this powerful oxidant will have to consider carefully the consequences of attempting to improve the election affinity by addition of axial ligands, which could lead to a loss in the orbital reorganization that is critical to increasing **Δ**_**acid**_.

## Heterogenous Catalysis Modeled With Periodic Boundary Conditions

### Catalytic Activity of Transition Metal Carbides

Transition metal carbides (TMCs) are a class of material known for their catalytic activity since 1973 (Levy and Boudart, 1973). These materials present different stoichiometries and structures depending on the position of the metal in the periodic table: Ti and Zr, on the left-hand side of the d-series, form stable and non-defective monocarbides, while metals toward the center of the periodic table present a lower carbon content, as seen in the widely studied case of Fe_3_C (Häglund et al., 1993; Oyama, 2008). All these materials, however, are considered valuable for industrial applications because of their relatively low cost, high durability and melting points as well as their catalytic activity (Hwu and Chen, 2005; Qi et al., 2013). TMCs have been tested for a wide variety of catalytic reactions, especially hydrogenation and dehydrogenation reactions for which their activity has proved to be qualitatively similar to that of Pt (Levy and Boudart, 1973; Delannoy et al., 2000). One such avenue of research, that exploits TMCs as catalysts for the hydrogen evolution reaction (HER), is particularly relevant to environmentally sustainable chemistry, as it is considered to be a key element in the transition from a fossil fuel-based to a hydrogen-based economy. The HER is the focus of a large amount of research interest worldwide for its role in alkaline water electrolysis, which produces highly pure H_2_, and in hydrogen fuel cells; both these applications make use of Pt as a catalyst to lower the overpotential required to perform the reaction down to appropriately 0.2 eV, but the cost and scarcity of the element (Yang, 2009), as well as the questionable environmental sustainability of Pt mining (Maboeta et al., 2006; Saurat and Bringezu, 2008; Glaister and Mudd, 2010) have driven the research toward catalysts composed of more earth-abundant elements such as TMCs. A related application, is the catalytic reduction of CO_2_ with H_2_, which usually aims at the production of CO or CH_3_OH, often requiring a surface-mediated proton transfer to transform CO_2_ into COOH (Posada-Pérez et al., 2017a).

**Figure 10:**
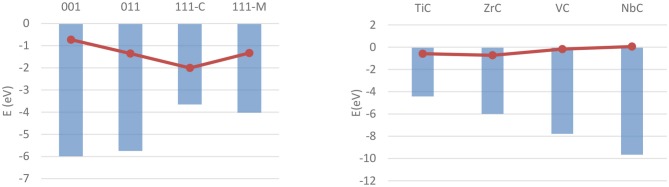
Correlation between ΔE_ads_ of a single hydrogen molecule (red dots) and the d-band centers (blue columns) on different carbides and surfaces. **(Left)** Shows the values for ZrC over the four investigated surfaces, **(Right)** shows the values for (001) surfaces over the four carbides.

The bulk and surface properties of TMCs have been well characterized in the past few years (Vines et al., 2005; Quesne et al., 2018), but fewer computational studies have been performed on their catalytic activity. Adsorption and activation studies have been performed for both H_2_ and CO_2_ for a wide range of early- and mid-series TMCs, with all of these studies modeling low-index surfaces of the catalysts using periodic boundary conditions. The (001) surfaces of MoC and Mo_2_C, the latter being either Mo- or C-terminated, have been the focus of a work from Posada-Pérez et al. on hydrogen adsorption (Posada-Pérez et al., 2017b), which found stable, dissociative adsorption of H_2_ on all three materials with no activation barrier when dispersion interaction correction is taken into account. The adsorption is found to occur primarily on top of surface carbon atoms on both materials, with adsorption energies calculated with the Perdew–Burke–Ernzerhof (PBE) (Perdew et al., 1996) functional in the −1 to −2.5 eV range, whilst consistently higher on the metal-rich carbide. Similarly, a study from Silveri et al. (2019) investigated the adsorption of H_2_ on TiC, VC, ZrC, and NbC, using a combination of periodic boundary conditions and the PBE functional, found adsorption to be exothermic on these systems' (001) surfaces as well. Unlike the former, however, this study was extended to the (011) and (111) surfaces as well, in order to obtain a more complete picture of the reactivity of the material. These data highlighted how the stability of the (001) surface is correlated with a lower reactivity on all carbides. More generally, all monocarbides show similar geometric and electronic properties of the adsorption, with the only major difference between the carbides being the strength of the adsorption in most cases. The exceptions are the carbon termination of the (111) surfaces of TiC and VC, which are found to be unstable in presence of hydrogen. However, the availability of adsorption energy data for all low-index surfaces across four carbides allowed these to be correlated with surface properties such as work function and *d-*band center position, as shown for the latter in Figure 10.

**Figure 11:**
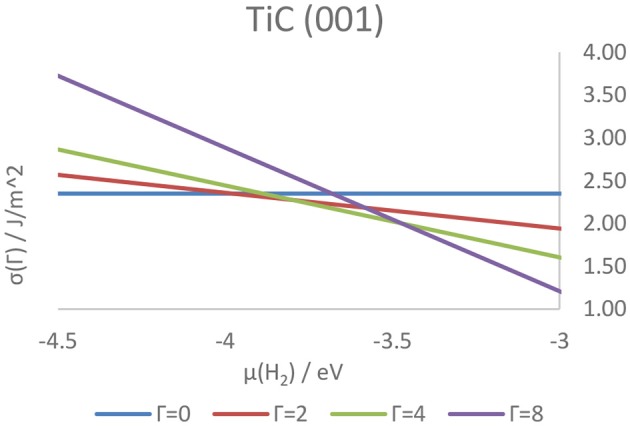
Surface free energies of surfaces in different hydrogenation states as a function of the chemical potential of gaseous hydrogen above the TiC (001) surface. The letter Γ indicates the number of atoms on the surface of the catalyst, with Γ = 0 and Γ = 8 corresponding, respectively to a coverage of 0 ML and 1 ML.

Higher coverage states have also been investigated, observing a decrease in the adsorption energy per atom as well as a similar, although not linear, decrease in work function, attributable to the electron transfer from the adsorbed hydrogens to the metallic slab. The coverage states of each surface of the four carbides were also predicted at a wide range of temperatures and pressures, and correlated with the tendency of the hydrogen either to adsorb on or desorb from the surface. As a result, it was shown how the strength of the C–H and M–H bonds on the (011) and (111) surfaces is predicted to hinder the feasibility of catalytic reactions such as HER on all higher-index surfaces. Conversely, the (001) surface - previously shown to be the lowest energy termination, shows a rapidly changing coverage state, suggesting its potential as an active termination for catalytic reactions involving a surface mediated hydrogenation and further elucidating the mechanistic details of the catalytic activity of the carbides. Figure 11 shows the hydrogen coverage states as a function of the H_2_ chemical potential for the TiC (001) surface.

MoC and Mo_2_C have also been studied computationally for their capability to adsorb CO_2_ and dissociate it to CO (Posada-Pérez et al., 2014). These studies show how both materials effectively activate carbon dioxide and in the case of the far more active Mo_2_C material, it is also possible to observe spontaneous dissociation. These studies, albeit not elucidating the catalytic behavior of early- and mid-series transition metal carbides completely, provide a powerful basis for further theoretical and experimental work on the catalytic activity of these materials for reactions such as HER, CO_2_ reduction and inverse water-gas shift and demonstrate the power of the periodic DFT approach to highlight fundamental properties of heterogenous catalyst. The elucidations of the mechanistic aspects of such reactions will help greatly in the development of the sustainable generation of fuels and chemicals as well as guiding the future design of the catalytic component of the hydrogen fuel cell—a challenge for which innovative catalysis is of paramount importance.

## Summary and Conclusions

The urgent need for society to move toward a greener and more sustainable future presents a very exciting opportunity for catalytic chemists. Many of the necessary changes in resource management and increased energy efficiency will be propelled by the directed design of new catalysts, for which a detailed theoretical understanding of the activity of current catalysts is a crucial part. Many very different computational techniques are being applied to the characterization of novel catalysts as a preliminary step to the engineering of new and much greener chemical route to important products. The implementation of a QM/MM protocol to the challenge of bioengineering the enzyme OleT_JE_, in order to increase its selectivity toward olefin production is explored in the first case study. This study indicates that the enzyme was able to effectively elevate a hydroxyl radical recombination barrier which leads to the alternative olefin pathway becoming competitive. This process is modulated by changes in the local solvation environment so there could be the potential to bioengineer an OleT isoenzyme to selectivity produce olefin for a sustainable route to bio-fuel production. The next case study used restricted cluster model calculations to investigate the ability of HOD to catalyze spin-forbidden oxygen activation. Interestingly, this study did not confirm the experimentally proposed reaction mechanism, but instead offered the potential for a novel green catalytic route for the activation of molecular oxygen via the stabilization of a triplet intermediate dioxygen species. The third case study explored the reactivity of a novel μ-nitrido-bridged diiron-oxo porphyrin that was able to catalyze the methane to methanol reaction under very mild conditions. This study used unrestricted DFT methods to determine that the acidity of the FeO anion was mostly responsible for its increased activity over the related mono-oxygen porphyrin catalysts. These results indicated that any improvement of the catalyst could not be made by sacrificing the novel orbital mixing along the Z-axis. Therefore, simply increasing the electron affinity of the FeO by binding a strong electron withdrawing group in the axial position is likely to be counterproductive. Finally, we consider several periodic DFT studies into the electronic properties and catalytic abilities of the low-index facets of early transition metal carbides. These studies point to the possibility of green catalytic routes toward the production of fuels and useful chemicals from the utilization of the green-house gas carbon dioxide; as well as the potential for these materials to be used as catalysts in hydrogen fuel cells.

## Author Contributions

All authors listed have made a substantial, direct and intellectual contribution to the work, and approved it for publication.

### Conflict of Interest Statement

The authors declare that the research was conducted in the absence of any commercial or financial relationships that could be construed as a potential conflict of interest.
